# Chimerism and immunological tolerance in solid organ transplantation

**DOI:** 10.1007/s00281-025-01052-x

**Published:** 2025-05-19

**Authors:** Birte Ohm, Anastasios D. Giannou, David Harriman, Jun Oh, Wolfgang Jungraithmayr, Dimitra E. Zazara

**Affiliations:** 1 Department of Thoracic Surgery, Faculty of Medicine, Medical Center – University of Freiburg, University of Freiburg, Freiburg, Germany; 2 Section of Molecular Immunology and Gastroenterology, I. Department of Medicine, University Medical Center Hamburg-Eppendorf, 20246 Hamburg, Germany; 3 Hamburg Center for Translational Immunology (HCTI), University Medical Center Hamburg-Eppendorf, 20246 Hamburg, Germany; 4 Department of General, Visceral and Thoracic Surgery, University Medical Center Hamburg-Eppendorf, Hamburg, Germany; 5 General Surgery, Liver, Pancreas and Intestinal Transplant Unit, Hospital Universitario-Fundación Favaloro, Buenos Aires, Argentina; 6 Department of Urologic Sciences, University of British Columbia, Vancouver, Canada; 7 University Children’s Hospital, University Medical Center Hamburg-Eppendorf, 20246 Hamburg, Germany; 8 Division of Thoracic Surgery, Rostock University Medical Center, Rostock, Germany; 9 Department of Thoracic Surgery, University Hospital Zurich, Zurich, Switzerland; 10 Division for Experimental Feto-Maternal Medicine, Department of Obstetrics and Fetal Medicine, University Medical Center Hamburg-Eppendorf, 20246 Hamburg, Germany

**Keywords:** Chimerism, Organ transplantation, Donor, Recipient, Tolerance, Feto-maternal microchimerism

## Abstract

In solid organ transplantation, chimerism inevitably occurs via the coexistence of donor-derived cells from the graft and host cells throughout the recipient. However, long-term immunosuppressive treatment is needed to suppress host immune responses to the foreign organ graft. The deliberate induction of stable mixed bone marrow chimerism to achieve donor-specific immunological tolerance in solid organ graft recipients is an ambitious goal that may significantly contribute to the long-term survival of solid organ grafts and their recipients. While this strategy has been effectively established in laboratory animals and some promising clinical case series have been reported, widespread clinical application is still limited by the toxicity of the necessary conditioning regimens. On the other hand, the naturally occurring chimeric state resulting from the bidirectional transplacental cell trafficking during pregnancy, the so-called feto-maternal microchimerism, can also induce immune tolerance and thus influence the outcome of mother-to-child or child-to-mother organ transplantation. This review provides an overview of the field's historical development, clinical results, and underlying principles of (micro) chimerism-based tolerance.

## Introduction

### Chimerism and the concept of immune tolerance

Long-term success after solid organ transplantation depends on the graft’s acceptance, which requires the recipient’s immune system to be unresponsive to donor antigens. Currently, long-term intake of immunosuppressive drug regimens, often consisting of multiple substances, is necessary to prevent acute and chronic rejections. However, long-term outcomes after solid organ transplantation remain limited by chronic graft rejection and the adverse effects of life-long immunosuppressive therapy, including risks of infection, cancer, drug toxicity, cardiovascular and metabolic disease.

The immunologic consequences of chimerism have been of interest in solid organ transplantation since seminal observations in dizygotic cattle demonstrated a specific acceptance of skin grafts from their sibling [1]. These observations led to the definition of immune tolerance, characterized by acceptance of the donor graft, rejection of third-party grafts, and the specific unresponsiveness of recipient immune cells to donor alloantigens without immunosuppressive treatment [2]. The induction of immune tolerance became the ultimate goal in solid organ transplantation as it would eliminate the requirement of immunosuppressive therapy while preventing immune-mediated graft rejection.

**Fig. 1:**
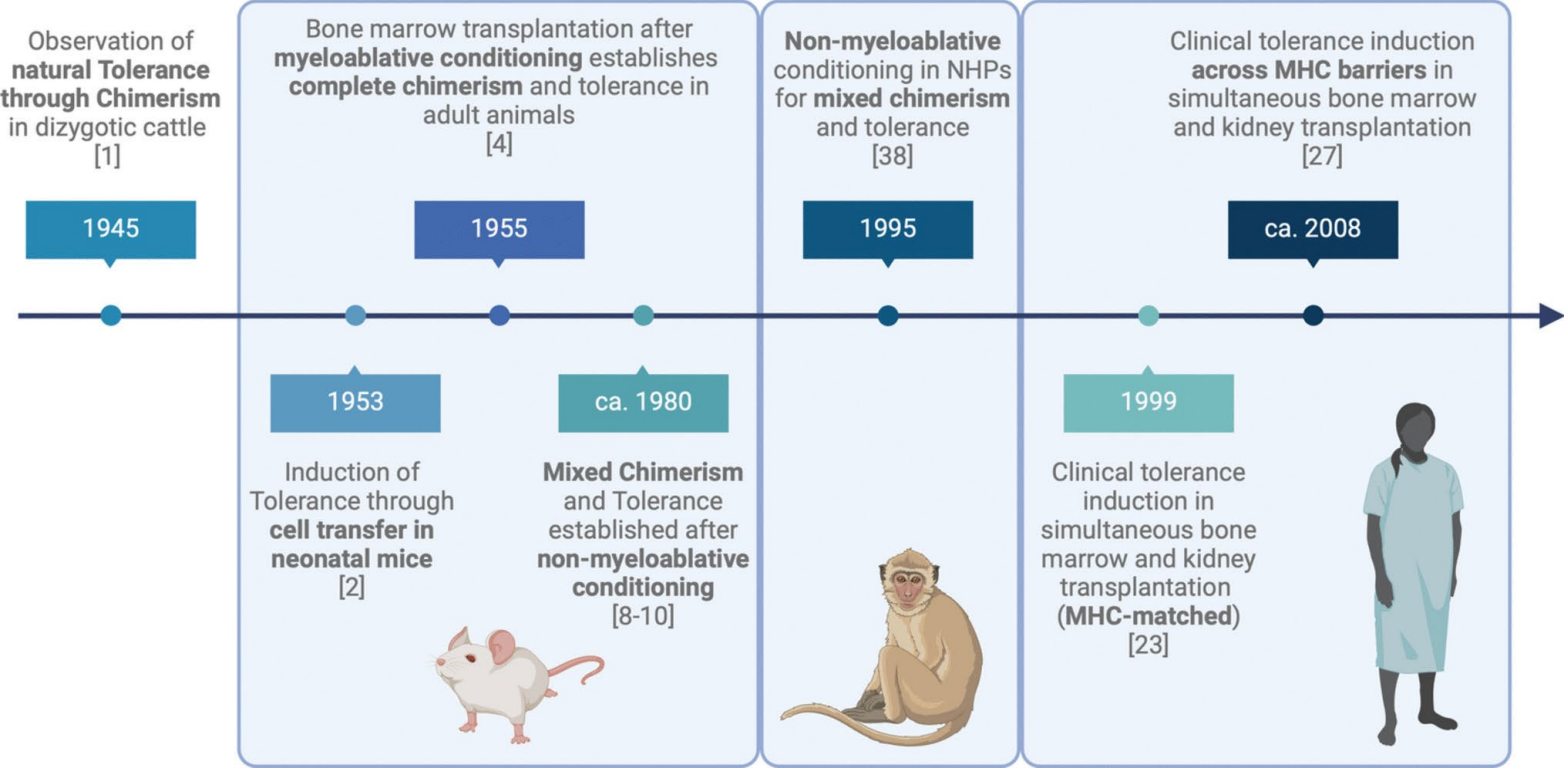
Milestones in preclinical and early clinical development of chimerism-based tolerance induction to solid organ grafts. NHPs = non-human primates

Subsequently, the principles of alloimmunity have been extensively researched to understand the mechanisms of chimerism induced by allotransplantation and to develop strategies to induce chimerism-based immune tolerance in the clinical setting. Figure 1 demonstrates the historical timeline, highlighting milestones in the preclinical and early clinical development of chimerism-based graft tolerance.

## Establishment of chimerism for tolerance induction to solid organ grafts in preclinical studies

### Rodent studies paved the way for the establishment of post-transplant chimerism-based immune tolerance and long-term allograft acceptance

The first intentional establishment of immune tolerance by induction of chimerism was reported in 1953 by Billingham et al. Neonatal mice were injected with bone marrow cells from another strain. At adult age, the animals accepted skin grafts from the bone marrow donor strain while rejecting third-party grafts [2]. The resulting animals are an example of mixed chimerism, defined by the presence of a mixture of two or more hematopoietic and immune cell lineages in the recipient’s bone marrow and lymphoid tissues. The extent of donor chimerism can vary greatly in mixed chimeras and can be determined by flow cytometry or PCR techniques. The presence of donor-derived cells at a frequency of > 1% to < 100% is termed macrochimerism. Microchimerism is defined as the presence of donor-derived cells at a lower frequency (< 1%) and can only be detected by PCR. Mixed chimaeras with macrochimerism demonstrate true immune tolerance as they show specific unresponsiveness to both donor and recipient alloantigens while rejecting third-party grafts. However, tolerance could only be induced by bone marrow infusion during the first few days after birth. Later infusions would result in no effect or enhanced immune responsiveness and accelerated graft rejection [3]. Even though the use of the protocol was not effective in adult animals, the study provided the first evidence that the immune system could be deliberately manipulated to become tolerant to alloantigens.

Allogeneic bone marrow infusions to unconditioned immunocompetent adult recipients are rapidly rejected and do not engraft. Based on this logic, bone marrow transfer requires prior depletion of the recipient immune system. Therefore, adult mice were next conditioned with lethal doses of total body irradiation before bone marrow transfer [4]. Instead of mixed chimerism, protocols using myeloablative irradiation induce complete chimerism. Complete chimerism refers to a state where recipient hematopoietic cells are entirely replaced by cells of donor origin. Complete chimaeras also accept solid organ grafts from their bone marrow donors and show specific unresponsiveness to these grafts [5]. Mechanistically, this kind of graft acceptance reflects the acceptance of self-antigens by the transplanted donor bone marrow rather than true organ transplant tolerance. Complete chimerism is desirable in patients receiving bone marrow transplantation as treatment for hematologic disease to prevent tumor recurrence. However, complete chimaeras are at risk of developing graft-vs-host disease (GvHD) as the bone marrow graft is prone to mounting immune responses against recipient alloantigens.

Irradiated animals receiving F1 hybrid bone marrow transplants accepted skin grafts from the allogeneic parental strain [6]. Using F1 hybrid bone marrow donors facilitated graft acceptance and prevented GvHD since the grafted bone marrow shared the alloantigens of both donor and host strains. Skin graft acceptance in these animals can be attributed to a failure of the F1 hybrid bone marrow to reject an organ of a parental strain. Thus, graft tolerance is merely a consequence of self-tolerance rather than immunological tolerance in the narrower sense.

Myeloablative conditioning is not suitable for organ transplant candidates due to its association with possibly life-threatening side effects, such as GvHD or profound leukopenia. The risk of these toxicities would be vastly disproportionate to the comparatively safe immunosuppressive treatment. Therefore, non-myeloablative strategies were employed next. Furthermore, bone marrow is usually transplanted in an MHC-matched setting while solid organ transplantation is routinely performed across MHC barriers. It is, therefore, necessary to define a conditioning regimen sufficient to permit the engraftment of fully mismatched bone marrow while avoiding unacceptable toxicity in the recipient.

Total lymphoid irradiation (TLI), targeting the spleen, thymus and lymph nodes, had initially been developed to treat Hodgkins Lymphoma [7]. A similar regimen was adopted in rodent transplantation models. Interestingly, adult TLI-treated mice given allogeneic bone marrow transplants demonstrated stable and self-perpetuating mixed chimerism without any evidence of GvHD [8–10]. Transplantation of skin grafts from the bone marrow donor and third-party strains revealed the specific acceptance of bone marrow donor grafts for up to 6 months [11]. In the mixed leukocyte reaction, isolated immune cells from mixed chimaeras were specifically unresponsive to donor alloantigens [12, 13].

After the successful application of TLI-based induction of stable mixed chimerism in mice, the principle was applied in rats which received intravenous MHC-mismatched bone marrow infusions and anti-thymocyte globulin (ATG) for tolerance induction in a heterotopic heart transplantation model.

Pre-transplant conditioning with TLI and ATG resulted in mixed chimerism and graft tolerance [11]. However, clinical translation of such a pre-transplant protocol would be impractical as the timing of donor organ availability is often uncertain in the clinical setting. Thus, the conditioning protocol was adapted for further studies and TLI/ATG was administered the day after organ transplantation. Donor bone marrow was then transferred twelve days after the organ transplantation. In this post-transplant regimen, rat heart allografts were not rejected during a follow-up period of 6 months [14]. The addition of post-transplant immunosuppressive treatment with cyclosporine further enhanced the establishment of stable mixed chimerism and transplant tolerance in the model [15].

Further studies in the rat model of heterotopic heart transplantation evaluated purified donor peripheral blood mononuclear cells (PBMCs) as a substitute for bone marrow cell infusion, as these would be easier to obtain for clinical translation. While the use of peripheral cells prolonged graft survival compared to control animals that did not receive any donor cell infusions, heart grafts showed long-term histologic evidence of chronic rejection [16]. Stable mixed chimerism and long-term graft acceptance were only achieved in animals receiving donor bone marrow infusions [16].

Stable mixed chimerism can successfully be established in rodents using a post-transplant regimen of total lymphoid irradiation, application of anti-thymocyte globulin, and donor bone marrow transplantation. Rodent stable mixed chimaeras show long-term graft acceptance without any risk of GvHD.

### Transient mixed chimerism is sufficient for long-term graft acceptance in large animal models

Subsequently, protocols involving bone marrow transplantation to induce immunological tolerance were studied in large animals, such as dogs, mini-swine, and non-human primates (NHPs). Low-dose total body irradiation (TBI) and bone marrow transplantation resulted in stable mixed chimerism persisting for several years in fully MHC-matched dogs. The animals received donor renal allografts and bilateral native nephrectomy with a short course of immunosuppression. At 5-year follow-up, all recipients showed normal renal function and no evidence of acute or chronic rejection [17]. Interestingly, the depletion of donor chimeric cells by another TBI and recipient leukocyte infusion led to continued acceptance of organ grafts, suggesting that the persistence of mixed chimerism is not necessary for graft tolerance in fully MHC-matched recipients [18].

In MHC-mismatched NHPs, non-myeloablative pre-transplant TBI, thymic radiation and ATG infusion resulted in mixed chimerism after combined bone marrow and kidney transplantation. Maintenance immunosuppression was not required in most recipient animals. In contrast to the observations in rodents, a loss of mixed chimerism was observed in NHPs after a few weeks. Regardless, renal allografts retained good function without immunosuppressive treatment [19].

In large animals, stable mixed chimerism was observed only in MHC-matched transplantation [17, 20], suggesting that its stability depends on MHC matching. Interestingly, stability of mixed chimerism was not required in MHC-mismatched transplantation for the long-term acceptance of kidney grafts.

## Clinical protocols for tolerance induction

**Fig. 2:**
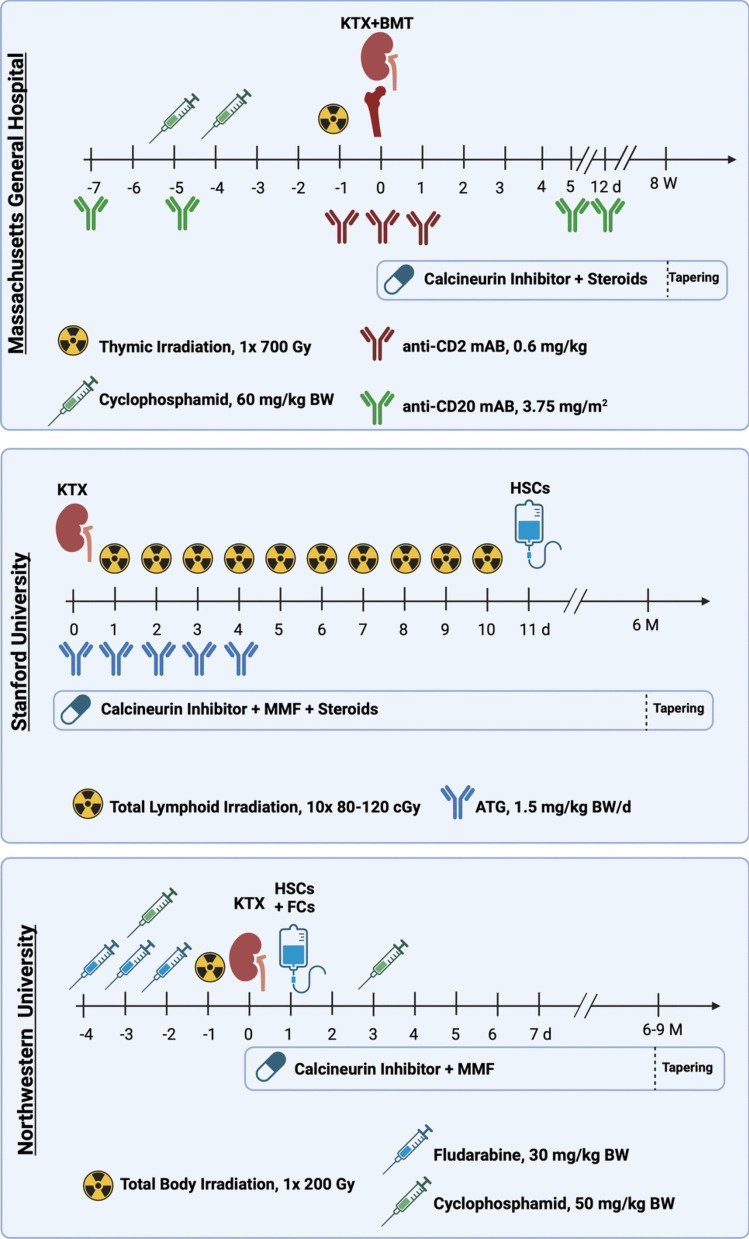
Overview of clinical protocols for tolerance induction in kidney transplantation. KTX = kidney transplantation, BMT = bone marrow transplantation, HSCs = hematopoietic stem cells, FCs = facilitating cells, mAB = monoclonal antibody, ATG = anti-thymocyte globulin, MMF = mycophenolate mofetil

Ideally, a tolerance protocol would preserve long-term graft function without posing a larger risk than immunosuppression therapy. Clinical organ transplant tolerance is termed operational tolerance, which is defined by the persistence of normal graft function and the absence of acute or chronic rejection without the intake of immunosuppressive drugs [21]. While several other centers have also tried to establish tolerance to organ grafts by induction of chimerism, three particular centers in living-donor kidney transplantation pioneered the clinical application of the concept [22]. An overview of the timeline, including the different regimens, drugs and treatments given to patients at the three facilities is depicted in Fig. 2.

### Massachusetts General Hospital

The first clinical trial of combined kidney and bone marrow transplantation for deliberate induction of immunological tolerance through chimerism was started in patients suffering from end-stage renal failure due to multiple myeloma [23]. These patients were neither eligible for conventional kidney transplantation due to their underlying malignant condition nor for isolated bone marrow transplantation due to their dependency on dialysis. Patients underwent a pretransplant conditioning regimen of cyclophosphamide, anti-thymocyte globulin and thymic irradiation and received combined kidney and bone marrow transplantation from an HLA-matched sibling [24]. The inclusion of cyclophosphamide was inspired by murine tolerance induction protocols where cytoreductive cyclophosphamide treatment was successfully used to replace low-dose total body irradiation [25, 26]. Patients were subsequently treated with cyclosporine A monotherapy, followed by attempts to wean immunosuppression [24]. While the protocol induced measurable chimerism in all ten patients enrolled, stable mixed chimerism was achieved in only one individual. Mixed chimerism was transient in five patients; the four remaining recipients showed complete chimerism. Graft acceptance was achieved in 50% of all patients enrolled who remained without any immunosuppressive treatment for up to 17 years after transplantation [24].

A cohort of five patients next received haploidentical bone marrow and kidney grafts after preconditioning with cyclophosphamide, an anti-CD2 antibody, and thymic irradiation [27]. Transient chimerism was induced in all patients. However, one patient developed acute rejection. The remaining four patients were successfully weaned from maintenance cyclosporine A treatment and demonstrated stable graft function for up to four years [27].

The preconditioning protocol was then changed to total body irradiation and fludarabine treatment for haploidentical donor-recipient pairs. Reduction of the pretransplant cyclophosphamide dose allowed for the addition of posttransplant treatment to prevent GvHD after transplantation. This scheme resulted in complete donor chimerism in 5 out of 6 patients (83.3%), and immunosuppression was successfully withdrawn in 3 patients (50%) [28]. The main adverse outcome of these trials was graft-vs-host disease: Overall, 70% of HLA-matched and 50% of haploidentical recipients developed a form of clinical GvHD. Among the ten HLA-matched recipients, three patients developed acute GvHD and five patients developed a form of chronic GvHD [24]. Among the six haploidentically transplanted recipients, two patients developed Grade 1 acute GvHD. There was no GvHD Grade 2–4. One patient developed chronic GvHD. Risking GvHD would be unacceptable in candidates for renal transplantation who do not suffer from hematologic malignancy.

The group employed another conditioning scheme for these kidney transplant candidates, consisting of pretransplant thymic irradiation, an anti-CD2 monoclonal antibody, and peritransplant rituximab induction [29]. Treatment with the anti-CD2 monoclonal antibody effectively targets effector memory T cells, which express higher levels of CD2 compared to naïve or regulatory T cells [30, 31]. With this treatment protocol, all recipients developed mixed chimerism for up to two weeks [29]. However, only four out of ten patients (40%) remained without immunosuppressive treatment at 11 to 18-year follow-up. No cases of GvHD were reported [29].

### Stanford University

At Stanford University, the feasibility of non-myeloablative conditioning regimens was first evaluated in patients with hematologic malignancy who were not considered fit for myeloablative conditioning due to comorbidity [32]. Persistent mixed chimerism was established in almost all recipients who had received G-CSF-mobilised peripheral blood mononuclear cells from HLA-matched donors [32].

Subsequently, the protocol was adopted to apply in combined MHC-mismatched living-donor kidney and hematopoietic cell transplantation. Notably, a post-transplant regimen was used for tolerance induction. After total lymphoid irradiation and application of anti-thymocyte globulin, peripheral mobilised CD24 + hematopoietic stem cells were infused on day 11 after kidney transplantation [33]. Maintenance immunosuppression consisted of prednisolone and cyclosporine A. In this first study, two out of six recipients (33.3%) developed mixed chimerism and were weaned from immunosuppression. However, immune tolerance was not achieved, as both participants developed cellular rejection a few months later [33].

In another cohort, 29 patients received HLA-identical transplantations and a modified conditioning regimen, including T cell or stem cell infusion [34, 35]. Out of 29 patients enrolled, 24 (82.7%) developed mixed chimerism for at least six months and were successfully weaned from immunosuppression. At the 15-year follow-up, no evidence of rejection was found in 22 patients (75.9%) without immunosuppressive treatment. The other two patients were returned to standard immunosuppression when they presented with evidence of rejection about four years after transplantation. Mixed chimerism was eventually lost in 19 of the 24 patients initially withdrawn from immunosuppression. Interestingly, graft function remained stable in most patients weaned from immunosuppression, even when mixed chimerism was lost [34, 35].

Another 22 recipients at Stanford University received HLA-mismatched grafts with a modified conditioning regimen transmitting more T cells and maintenance immunosuppression with mycophenolate mofetil and tacrolimus [35]. Successful induction of mixed chimerism was observed in 15 recipients (68.2%), and eleven patients were subsequently weaned from mycophenolate mofetil as they did not show evidence of rejection or GvHD. In ten recipients, chimerism persisted, and nine of those patients were weaned from tacrolimus. However, immunosuppression had to be taken up again by all recipients, as chimerism was lost when subtherapeutic levels of tacrolimus were reached, and some recipients developed rejection [35].

The Stanford protocols successfully induced tolerance in most recipients after MHC-matched transplantation without the need for pre-transplant conditioning. Even though loss of chimerism was common, graft function remained stable in MHC-matched transplantation. In contrast, in a MHC-mismatched setting, all patients remained dependent on some immunosuppressive treatment. As there were no differences in chimerism levels between MHC-matched and -mismatched settings, the persistence of chimerism is not the critical factor for the induction of tolerance. It is likely that the Stanford protocol influences peripheral immunoregulation sufficiently to control alloimmunity in HLA-matched transplantation but not across HLA barriers.

### Northwestern University

Pre-transplant conditioning with cyclophosphamide, fludarabine and non-myeloablative total body irradiation is used at Northwestern University. The day after kidney transplantation, patients receive a cell product derived from mobilised hematopoietic stem cells enriched in a specific CD8^+^ TCR^−^ cell population [36]. These so-called “facilitating cells” (FC) were first described in 1999 to promote the engraftment of hematopoietic stem cells across MHC barriers [37]. The clinically used cell product primarily consists of immature plasmacytoid dendritic cells [36]. Mycophenolate mofetil and tacrolimus were used for maintenance immunosuppression and discontinued one year after transplantation when persistent chimerism and allograft tolerance were confirmed. Eight patients underwent the regimen initially and complete chimerism persisted in five patients (62.5%) for at least a year [36].

In a Phase II trial, 37 patients underwent the Northwestern protocol in HLA-mismatched combined kidney and stem cell transplantation. Induction of chimerism was successful in 35 patients (94.5%) and persisted in 26 (70.3%). After immunosuppression withdrawal, all patients with persistent chimerism remain in stable clinical condition with preserved graft function [38]. GvHD occurred in one participant not weaned from immunosuppression [38]. Subsequently, a phase III trial aimed to further evaluate the use of the FC therapy was launched (FREEDOM-1; NCT03995901). Safety concerns were raised after one participant died from acute GvHD, causing a temporary pause to the trial. More recently, the study was prematurely terminated by the sponsor.

Taken together, immunological tolerance toward organ grafts through chimerism can be achieved in humans, and long-term withdrawal from immunosuppression is possible. However, the promising results from preclinical studies in rodents and large animals are not fully reflected in humans. In some cases, complete chimerism develops, putting patients at significant risk of GvHD. Once established, mixed chimerism is frequently lost over time. Interestingly, the stability of mixed chimerism is not a necessary precondition for long-term clinical operational tolerance.

## Mechanisms of immune tolerance in mixed chimeras

The key goal is to reach stable mixed chimerism by using conditioning regimens sufficient for the engraftment of fully mismatched bone marrow without causing unacceptable toxicity in the recipient. The lack of non-toxic conditioning protocols remains the main barrier to widespread application.

The establishment of mixed chimerism is desirable as it places a self-replenishing source of donor antigen-presenting cells in the recipient, extending their mechanisms of self-tolerance to include the donor organ. Appropriate conditioning regimens would allow donor hematopoietic stem cells to engraft in the recipient bone marrow while pools of self-renewing recipient hematopoietic stem cells continue to co-exist with the bone marrow graft. This way, the stem cell pools give rise to a mixture of hematopoietic cell lineages of recipient and donor origin.

**Fig. 3:**
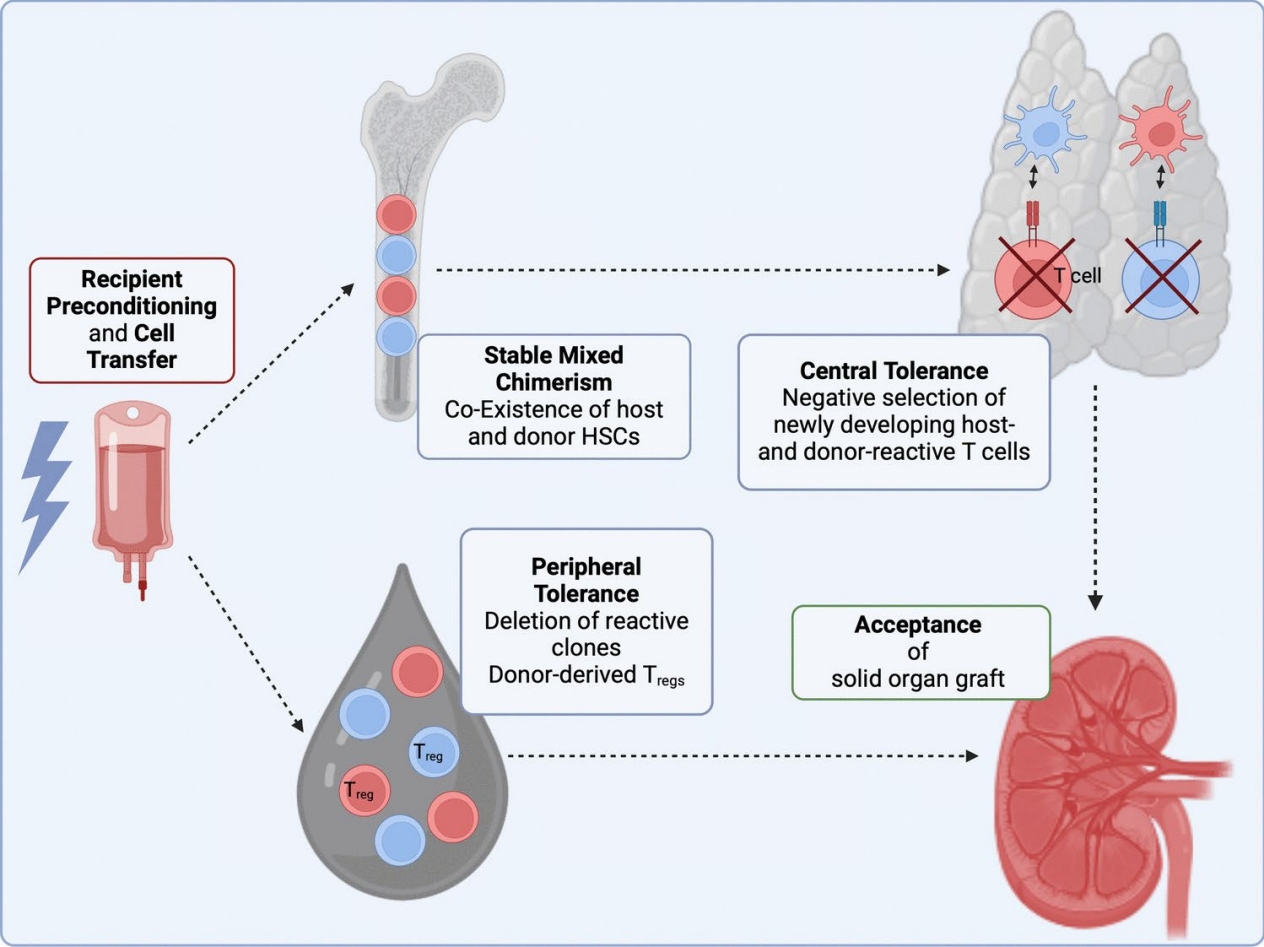
Mechanism of tolerance induction in mixed chimerism. Donor-derived cells and tissues are depicted in red, host-derived cells in blue. After recipient preconditioning and the transfer of donor-derived hematopoietic stem cells (HSCs), both donor- and host-derived cells co-exist in the recipient bone marrow. Engraftment of donor-derived dendritic cells in the recipient thymus enables the mutual negative selection of newly developing host- and donor-reactive T cells. Peripheral tolerance mechanisms, such as the deletion of host- and donor-reactive T cell clones and the presence of regulatory T cells (T_reg_) of both donor and recipient origin, also contribute to the long-term acceptance of the donor organ

Tolerance induction by mixed chimerism depends on both central and peripheral tolerance mechanisms. The following paragraphs present the mechanistic base of tolerance by chimerism and Fig. 3 gives an overview of the concept. Current barriers to stable mixed chimerism are presented along with the efforts to overcome them therapeutically.

### Bi-directional intra-thymic T cell selection confers central tolerance in stable mixed chimaeras

In persistent mixed chimerism, donor-derived antigen-presenting cells (APCs) are engrafted in the recipient thymus [12, 39, 40]. These donor-derived APCs probably derive from transmitted progenitor cells or from peripheral dendritic cells migrating into the thymus [41–43].

Donor APCs in the thymus then present donor antigens to the developing host T cells, inducing the deletion of alloreactive host-vs-graft T cells [12]. Vice versa, host-derived APCs present host self-antigens to the transmitted donor T cells, causing the deletion of graft-vs-host reactive T cells. This way, a bi-directional process of clonal deletion is established, conferring central tolerance. Central tolerance is the principal mechanism inducing and maintaining immune tolerance when recipient T cells are globally depleted before transplantation [44]. Transmitted donor T cells are a potential source of antigen for the induction of central tolerance, as peripheral T cells can re-enter the thymus. In mice, transfer of donor T cells has been shown to induce tolerance across MHC barriers [45].

Even though thymopoiesis diminishes with age, some function is maintained lifelong [46]. Indeed, thymic output is a major contributor to T cell reconstitution after antiretroviral therapy in the context of HIV infection and after hematopoietic stem cell transplantation, even in aged patients [47, 48]. Mechanisms for ongoing tolerization of newly developing donor-reactive T cells are necessary for robust and lasting tolerance. Tolerance protocols exclusively relying on peripheral mechanisms are hampered by a continuous output of donor-reactive T cells from the thymus [49]. On the other hand, relying on central tolerance alone is insufficient to allow immunosuppression removal without risking organ graft rejection or graft-vs-host reactions. Mixed chimerism induced by low-intensity host conditioning leaves residual host post-thymic T cells, potentially mediating organ rejection. Likewise, post-thymic donor T cells accompany the hematopoietic graft and their persistence can confer graft-vs-host reactions. Furthermore, some solid organ grafts, such as the liver or intestine, carry large numbers of post-thymic donor lymphocytes with potential graft-vs-host reactivity. Therefore, peripheral immunoregulatory mechanisms are also needed to control bidirectional alloreactivity.

### Peripheral T cell deletion can stabilize mixed chimerism and graft acceptance

Peripheral T cell functions can be controlled by a progressive deletion of T cell clones or by the induction of unresponsive states such as T cell anergy or exhaustion. Longitudinal studies demonstrated that donor-reactive T cell clones are progressively deleted in blood and biopsy samples of tolerant individuals after solid organ transplantation [50, 51]. Persistent peripheral T cells likely mediate rejection in non-tolerant individuals. This barrier to immune tolerance may be overcome by pharmacological enhancement of peripheral T cell deletion.

In mice, it was shown that T cell depletion by monoclonal anti-CD4 and anti-CD8 antibody therapy contributed to the induction of mixed chimerism [52].

Physiologically, peripheral T cell deletion can be triggered by the receptor-triggered extrinsic or the mitochondria-dependent intrinsic pathway. The extrinsic pathway is initiated by Fas-FasL interaction and facilitates the deletion of T cells after contact with their cognate antigen, resulting in activation-induced cell death. In contrast, the intrinsic pathway is controlled by members of the Bcl-2 family of apoptotic regulators. In mice, applying a Bcl-2/Bcl-X_L_ inhibitor combined with costimulatory blockage allowed for the induction of mixed chimerism after donor bone marrow infusion, even without previous lymphoablative conditioning [53]. Recently, Bcl-2 inhibition was found to promote stable mixed chimerism and long-term renal graft survival in non-human primates after total body irradiation conditioning [54].

In contrast to laboratory rodents, long-term stable mixed chimerism is far more challenging to achieve in large outbred animals and humans. These differences can be explained by the presence of peripheral memory T cells and cross-reactive T cells representing a barrier to immune tolerance. Indeed, non-human primates with high frequencies of pre-transplant donor-reactive memory T cells fail to become tolerant after non-myeloablative combined kidney and bone marrow transplantation [55]. Cross-reactive T-cell clones can originate from prior infections. For example, such heterologous immunity has been demonstrated in anti-CMV T cell clones, where up to 45% of clones have alloreactive properties [56].

Strategies to eliminate donor-reactive memory T cells have been explored in NHPs and show promising results. The fusion protein Alefacept targeting LFA-3/CD2 interactions selectively depleted effector memory T cells, leading to stable mixed chimerism, and long-term survival of renal allografts after a post-transplant conditioning regimen [57]. Similarly, the depletion of CD8 + memory T cells by application of a humanised anti-CD8 monoclonal antibody achieved mixed chimerism and survival of renal grafts in most NHP recipients [58].

### Peripheral regulatory T cell populations promote chimerism and graft tolerance

In contrast to peripheral allo-effector T cells, the presence of peripheral regulatory T cell (Treg) populations is associated with graft tolerance. Indeed, an increased frequency of memory regulatory T cells is found in kidney graft recipients who developed spontaneous operational tolerance [59]. An enrichment of Tregs has been observed early after transplantation in patients treated with the non-myeloablative protocol at Massachusetts General Hospital [60]. Tolerant patients demonstrated an enrichment of donor-reactive Tregs specifically [61]. Expansion of donor-specific Tregs was found in NHPs that were tolerant to solid organ grafts after undergoing non-myeloablative conditioning regimens [62]. In line with these findings, deletion of donor-reactive Tregs in a murine model disrupts previously established tolerance [63]. Furthermore, Tregs can promote infectious tolerance to a broader diversity of donor antigens by converting conventional T cells to induced regulatory T cells [63, 64].

Pharmacologically, Treg expansion can be promoted by subcutaneous injection of Interleukin-2 (IL-2), as demonstrated in chronic GvHD [65]. However, IL-2 also activates effector T cells, leading to graft rejection in previously tolerant NHPs with mixed chimerism [66].

Peripheral Tregs can be conserved by costimulatory blockage, a strategy often used for tolerance induction in preclinical studies. In contrast to conventional effector T cells, Tregs do not upregulate CD40L upon activation [67], suggesting that CD40/CD40L blockage can increase Treg frequency. In line with this, anti-CD28 antibody treatment augmented peripheral and intragraft Treg frequencies in NHPs after kidney and heart transplantation [68]. Furthermore, co-stimulatory blockage is a promising strategy as it also leads to the deletion of peripheral effector T cells [69].

Instead of promoting an endogenous enhancement of Treg frequency, Tregs could be applied as ex vivo manufactured cell products. In rodent models, the direct administration of polyclonal Tregs, in addition to donor bone marrow, promoted the establishment of stable chimerism and tolerance [70]. Applying allospecific Tregs might even be advantageous over polyclonal cell products to induce tolerance [71]. In another preclinical study, allogeneic MHC-targeted chimeric antigen receptors were used to redirect Treg specificity to the allograft [72]. Even though the ex vivo manufacturing of allospecific Treg products for clinical application is difficult due to their low frequency, polyclonal Treg products are currently evaluated in clinical trials for GvHD prophylaxis and in kidney and liver transplantation [73–75].

### Donor lymphoid cells from solid organ grafts can promote chimerism and counteract alloimmunity

Donor lymphoid cells can not only be brought into the recipient by bone marrow transfer, but also by the solid organ graft itself. Especially, intestinal and hepatic grafts harbor high amounts of donor lymphoid cells. Interestingly, recipients of intestinal allografts commonly show mixed chimerism of peripheral leukocytes without evidence of neither GvHD nor graft rejection [50]. This is also observed after multivisceral transplantation, which includes the donor stomach, pancreas, and liver in addition to the small intestine [76]. Such grafts are rich in donor leukocytes in the mucosal lymphoid tissues, lymph nodes, lymphoid follicles, and Peyer’s patches. Therefore, it seems likely that donor-derived immune cells introduced to the recipient with the allograft mount anti-host responses, balancing out host-vs-graft (HvG) alloimmune reactions without causing GvHD.

In rodents, it has been shown that infusions of donor lymphocytes can convert mixed chimerism to full donor chimerism, based on graft-vs-host (GvH) reactivity [77]. In these animals, the alloresponse remains confined to the lymphohematopoietic system and does not involve epithelial tissues, resulting in the absence of GvHD [77]. Imaging studies of T cell migration later showed that tissue inflammation is a prerequisite for the migration of graft-vs-host reactive T cells to epithelial targets [78].

GvH-reactive donor leukocytes promote tolerance through their local action at the solid organ graft and on bone marrow level. Locally, an expansion of GvH-reactive T cells in the intestinal mucosa of the graft is associated with a rapid replacement of donor myeloid cells by APCs of recipient origin [50]. This way, an increased ratio of GvH-reactive to HvG-reactive T cells is present in the allograft, facilitating tolerance. The GvH-reactive cells are believed to be derived from tissue-resident memory cells which are cross-reacting to alloantigens [79].

In the bone marrow of recipients of intestinal grafts, donor-derived T cells and even hematopoietic stem and progenitor cells can be found even after 100 days after transplantation [80]. It was demonstrated that some of the T cell clones that were found in the bone marrow were present in the graft mucosa at earlier time points, suggesting that tissue-resident GvH-reactive cells migrate to the bone marrow and facilitate the engraftment of donor cells by cytolysis of recipient hematopoietic cells [80].

Taken together, tissue-resident donor lymphoid cells can promote multilineage mixed chimerism and tolerance without the risk of GvHD by GvH reactivity that is confined to the lymphohematopoietic system. Clinically, these reactions are most prominent in recipients of intestinal and multivisceral allografts.

## Feto-maternal microchimerism as natural determinant of transplantation tolerance

**Fig. 4:**
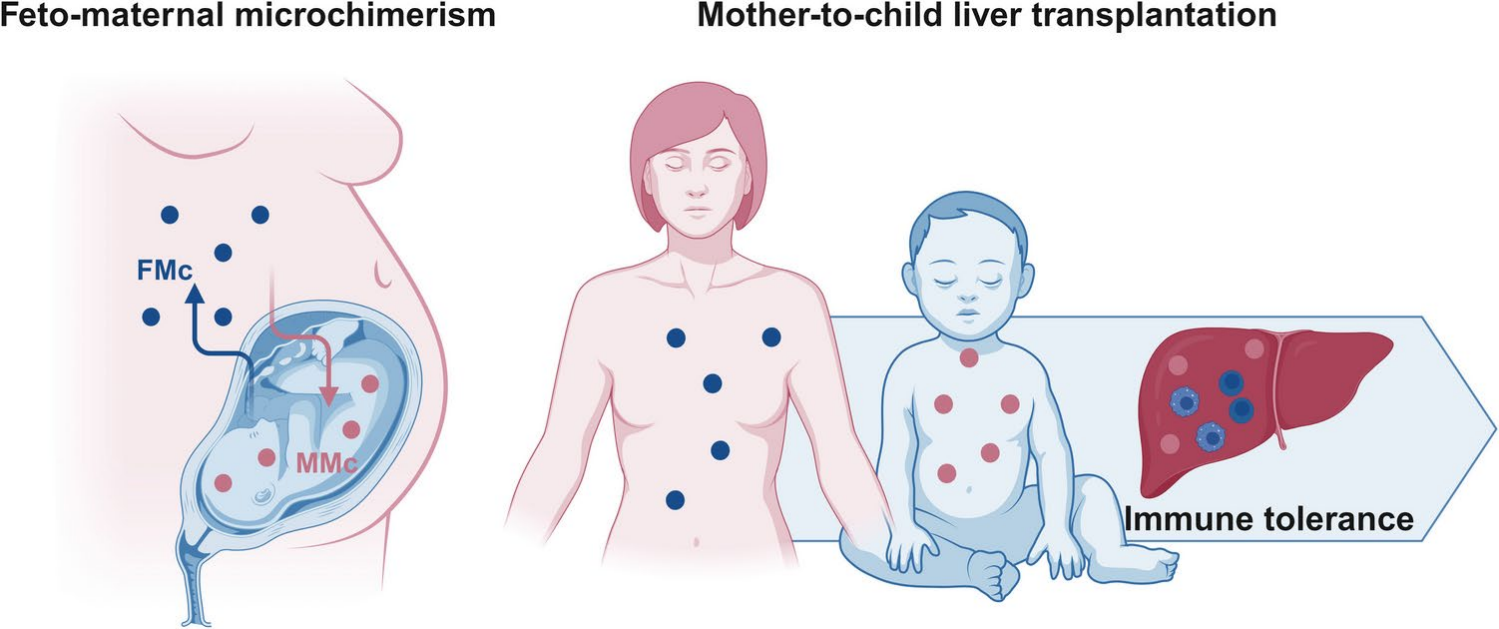
Feto-maternal microchimerism and organ transplantation. The bidirectional transplacental cell trafficking during pregnancy shapes a microchimeric state for both mother and offspring, with maternal (MMc) and fetal microchimeric cells (FMc) residing in fetal and offspring tissues, respectively, for a long time after birth. Such an early and prolonged exposure of the offspring to these maternal cells promotes immune tolerance to maternal antigens, which may influence the transplantation outcome in cases of mother-to-child organ donation

Feto-maternal microchimerism refers to bidirectional transplacental cell trafficking during pregnancy, shaping a microchimeric state for both mother and offspring [81], known as fetal and maternal microchimerism, respectively (Fig. 4).

Maternal microchimerism encompasses the presence and persistence of maternal cells in various tissues and organs of the offspring [82–85]. Although several types of maternal microchimeric cells (MMc) have been identified—including progenitor cells, stem cells, differentiated immune cells, and somatic tissue-specific cells such as intrahepatic biliary epithelial cells and insulin-producing beta cells- the complete repertoire of MMc within individual fetuses remains incompletely characterized [82, 85–89]. Of note, evidence suggests that the composition of the MMc pool reaching the fetus may be influenced by several factors, including maternal–fetal HLA compatibility and pregnancy conditions such as maternal infection, inflammation and related immune activation, thereby resulting in inter-individual variability [83, 90, 91]. Due to their longevity in the offspring’s body and immunological effects, the potential role of MMc in organ transplantation outcomes has recently gained attention. Although MMc are genetically discordant and thus foreign to the offspring, they are not rejected by the offspring’s immune system [92]. In fact, an early and prolonged exposure of the offspring to these maternal cells attributes a degree of immune tolerance to maternal antigens. Of note, MMc have been shown to boost the production of tolerogenic fetal regulatory T cells that attenuate the fetal immune response to non-inherited maternal antigens (NIMA) [92]. Thus, it can be hypothesized that such tolerance persists over time and may determine the outcome of postnatal transplantation involving maternally-derived donor organs [85, 92]. Indeed, long-term follow-up of pediatric liver transplant recipients reveals clinical and immunological benefits of living donor liver transplantation, especially with maternal grafts [93]. Specifically, maternal living donor grafts have been linked with lower rates of organ failure and acute cellular rejection as well as lower maintenance immunosuppression requirements in pediatric transplant recipients with biliary atresia [94–97]. Interestingly, increased maternal cells have been found in the livers of children with biliary atresia and are considered crucial players in disease pathogenesis by fueling a graft-versus-host cascade of events in the liver [88, 98, 99]. However, in the case of transplantation, these cells seem to be beneficial for graft tolerance, since biliary atresia patients receiving a maternal liver exhibit improved outcomes compared to patients receiving a paternal liver [95]. Similar findings have also been reported in the case of kidney transplantation, with maternal living renal graft donation resulting in 50% lower treated rejection rate than paternal donation, and higher long-term graft survival in sibling recipients of kidney grafts expressing NIMAs [100–102]. Nevertheless, observations concerning kidney transplantation are in general ambiguous, with a few studies demonstrating no improvement in transplantation outcome of mother-to-child kidney grafts [103–105] and others conversely associating maternal renal donation with higher rejection and graft failure risk [106]. Such a lack of a favorable “NIMA” impact in the case of maternal kidney donation may be—at least partially- explained by the higher immunogenicity of renal compared to liver allografts [107, 108], which may cover any potential MMc-mediated tolerance. Importantly, a beneficial MMc effect has also been reported in cases of hematopoietic stem cell transplantation, with MMc immunomodulating effects resulting in attenuated GvHD incidence or severity and lower relapse rates following transplantation [109, 110]. Taken together, these observations suggest that maternal microchimerism can influence organ transplantation outcomes by modulating immunity and promoting immune tolerance, enhancing donor-recipient compatibility, and reducing the risk for rejection and graft failure. Further research is warranted in order to fully characterize the maternal microchimerism-related cellular legacy and its inter-individual variations, in order understand their impact on transplantation outcome and explore potential clinical applications in this context.

Leading to a similar immune priming, fetal microchimerism, comprising the presence and long-term persistence of fetal cells in maternal tissues, may also induce immune tolerance and thus, impact transplantation outcomes in mother-to-child or child-to-mother transplant donations [85]. The pool of fetal microchimeric cells (FMc) is likely more homogeneous than the MMc repertoire. Various FMc types have been identified, including T and B cells, monocytes and macrophages, NK cells, granulocytes, as well as hematopoietic and mesenchymal stem cells [85, 111]. Additionally, similar to maternal microchimerism, FMc composition may vary between individuals due to aforementioned factors. It can be thus hypothesized that fetal cells remaining in the mother’s body after pregnancy may act as tolerizing agents thereby rendering the maternal immune system less aggressive towards fetal antigens. Additionally, fetal cells have been found to promote the development of regulatory T cells, which are crucial for immune tolerance and immune suppression, and could therefore contribute to favorable outcomes in maternal recipients of offspring-derived organs [112]. Conversely, fetal microchimerism may also contribute to allorecognition and promote immune activation by priming the maternal immune system to react to fetal antigens, thereby resulting in chronic rejection of a transplanted organ carrying such antigens [113–115]. These contradictory effects seem to largely depend on a balance between tolerant allogeneic T-cell-mediated responses and proinflammatory allogeneic B-cell-based responses. In mice, allogeneic pregnancy results in rejection of offspring-matched allogeneic heart grafts transplanted in postpartum mice despite allo-specific Treg expansion. Interestingly, these grafts are tolerated in postpartum B-cell-deficient recipients, thereby indicating that pregnancy-triggered sensitization may surpass pregnancy-induced T-cell-mediated tolerance of offspring-derived grafts [113, 114]. Similarly, pregnancy-sensitized non-human primates receiving offspring-matched renal transplants required de-sensitization and belatacept-based maintenance along with conventional tacrolimus-based immunosuppression to achieve prolonged graft survival [115]. These observations suggest that female recipients of offspring-matched grafts may benefit from immunosuppression strategies targeting pregnancy-induced humoral immunity while sustaining the beneficial effects of pregnancy-triggered T-cell-mediated immune tolerance. However, similar to maternal microchimerism, more research is required to understand the implicated mechanisms and FMc variations, exerted effects and potential therapeutic applications of fetal microchimerism in organ transplantation.

## Conclusion

The establishment of mixed chimerism to achieve immunological tolerance is a promising concept which may provide substantial benefits to organ transplant recipients who are currently threatened by both the possibility of acute or chronic graft rejection and the side effects of long-term immunosuppressive drug therapy. However, the main hurdle to wide clinical application is the ongoing search for the ideal conditioning regimen, which must allow sufficient engraftment of donor bone marrow without exposing patients to unacceptable toxicity. While such non-toxic tolerance regimens have been successfully established in laboratory rodents, clinical translation still lags behind. Translation is hampered by distinct immunological differences, such as heterologous immunity between inbred laboratory rodents kept under specific pathogen-free conditions and outbred large animals or humans exposed to environmental influences. Several strategies to facilitate engraftment in non-myeloablative conditioning regimens have been researched in rodents. Currently, the lack of respective pharmacological agents for clinical application poses another translational hurdle. Improvements in the efficacy and safety of tolerance induction and a focus on post-transplantation regimens are necessary to pave the way towards routine clinical use in a wider variety of solid organ grafts and deceased donors.

In parallel, a better understanding of mechanisms involved in natural immune tolerance, as seen in the case of feto-maternal microchimerism may open up new therapeutic avenues and improve transplantation outcomes in mother-to-offspring and offspring-to-mother transplant donations. Insights into the protective and – to a lesser extent – detrimental implications of microchimerism in this context will allow the design of appropriate pre- and post-transplantation immunomodulatory and -suppression approaches in order to optimize transplantation outcome and prolong graft survival.

## Data Availability

The generated dataset is available from the corresponding author on reasonable request.

## References

[1] Owen RD (1945) Immunogenetic consequences of vascular anastomoses between bovine twins. Science 102:400–401. 10.1126/science.102.2651.400 17755278 10.1126/science.102.2651.400

[2] Billingham RE, Brent L, Medawar PB (1953) Actively acquired tolerance of foreign cells. Nature 172:603–606. 10.1038/172603a0 13099277 10.1038/172603a0

[3] Billingham RE, Brent L, Medawar PB (1956) Quantitative studies on tissue transplantation immunity. III. Actively acquired tolerance. Philos Trans R Soc Lond B Biol Sci 239:357–414

[4] Main JM, Prehn RT (1955) Successful skin homografts after the administration of high dosage X radiation and homologous bone marrow. JNCI: J Natl Cancer Inst 15:1023–1029. 10.1093/jnci/15.4.1023 13233946

[5] Dey B, Sykes M, Spitzer TR (1998) Outcomes of recipients of both bone marrow and solid organ transplants. A review. Medicine (Baltimore) 77:355–369. 10.1097/00005792-199809000-00005 9772924 10.1097/00005792-199809000-00005

[6] Billingham RE, Brent L, Medawar PB, Sparrow EM (1954) Quantitative studies on tissue transplantation immunity. I. The survival times of skin homografts exchanged between members of different inbred strains of mice. Proc R Soc Lond B Biol Sci 143:43–58. 10.1098/rspb.1954.0053 13224650 10.1098/rspb.1954.0053

[7] Sutherland DER, Ferguson RM, Simmons RL et al (1984) Total lymphoid irradiation. J Urol. 10.1016/S0022-5347(17)50288-8

[8] Slavin S, Strober S, Fuks Z, Kaplan HS (1976) Long-term survival of skin allografts in mice treated with fractionated total lymphoid irradiation. Science 193:1252–1254. 10.1126/science.785599 785599 10.1126/science.785599

[9] Slavin S, Strober S, Fuks Z, Kaplan HS (1977) Induction of specific tissue transplantation tolerance using fractionated total lymphoid irradiation in adult mice: long-term survival of allogeneic bone marrow and skin grafts. J Exp Med 146:34–48. 10.1084/jem.146.1.34 17647 10.1084/jem.146.1.34 PMC2180728

[10] Sharabi Y, Sachs DH (1989) Mixed chimerism and permanent specific transplantation tolerance induced by a nonlethal preparative regimen. J Exp Med 169:493–502. 10.1084/jem.169.2.493 2562984 10.1084/jem.169.2.493 PMC2189213

[11] Slavin S, Reitz B, Bieber CP et al (1978) Transplantation tolerance in adult rats using total lymphoid irradiation: permanent survival of skin, heart, and marrow allografts. J Exp Med 147:700–707. 10.1084/jem.147.3.700 147301 10.1084/jem.147.3.700 PMC2184184

[12] Tomita Y, Khan A, Sykes M (1994) Role of intrathymic clonal deletion and peripheral anergy in transplantation tolerance induced by bone marrow transplantation in mice conditioned with a nonmyeloablative regimen. J Immunol 153:1087–1098 8027542

[13] Higuchi M, Zeng D, Shizuru J et al (2002) Immune tolerance to combined organ and bone marrow transplants after fractionated lymphoid irradiation involves regulatory NK T cells and clonal deletion1. J Immunol 169:5564–5570. 10.4049/jimmunol.169.10.5564 12421933 10.4049/jimmunol.169.10.5564

[14] Woodley SL, Gurley KE, Hoffmann SL et al (1993) Induction of tolerance to heart allografts in rats using posttransplant total lymphoid irradiation and anti-T cell antibodies. Transplantation 56:1443–1447. 10.1097/00007890-199312000-00032 8279017 10.1097/00007890-199312000-00032

[15] Lan F, Hayamizu K, Strober S (2000) Cyclosporine facilitates chimeric and inhibits nonchimeric tolerance after posttransplant total lymphoid irradiation. Transplantation 69:649–655. 10.1097/00007890-200002270-00029 10708124 10.1097/00007890-200002270-00029

[16] Hayamizu K, Lan F, Huie P et al (1999) Comparison of chimeric acid and non-chimeric tolerance using posttransplant total lymphoid irradiation: cytokine expression and chronic rejection. Transplantation 68:1036–1044. 10.1097/00007890-199910150-00023 10532547 10.1097/00007890-199910150-00023

[17] Kuhr CS, Yunusov M, Sale G et al (2007) Long-term tolerance to kidney allografts in a preclinical canine model. Transplantation 84:545–547. 10.1097/01.tp.0000270325.84036.52 17713441 10.1097/01.tp.0000270325.84036.52

[18] Graves SS, Mathes DW, Georges GE et al (2012) Long-term tolerance to kidney allografts after induced rejection of donor hematopoietic chimerism in a preclinical canine model. Transplantation 94:562–568. 10.1097/TP.0b013e3182646bf1 22929594 10.1097/TP.0b013e3182646bf1 PMC3448791

[19] Kawai T, Poncelet A, Sachs DH et al (1999) Long-term outcome and alloantibody production in a non-myeloablative regimen for induction of renal allograft tolerance. Transplantation 68:1767–1775. 10.1097/00007890-199912150-00022 10609955 10.1097/00007890-199912150-00022

[20] Huang CA, Fuchimoto Y, Scheier-Dolberg R et al (2000) Stable mixed chimerism and tolerance using a nonmyeloablative preparative regimen in a large-animal model. J Clin Invest 105:173–181. 10.1172/JCI7913 10642595 10.1172/JCI7913 PMC377429

[21] Auchincloss H (2001) In Search of the Elusive Holy Grail: the Mechanisms and Prospects for Achieving Clinical Transplantation Tolerance. Am J Transplant 1:6–12. 10.1034/j.1600-6143.2001.010103.x 12095040 10.1034/j.1600-6143.2001.010103.x

[22] Annamalai C, Kute V, Sheridan C, Halawa A (2023) Hematopoietic cell-based and non-hematopoietic cell-based strategies for immune tolerance induction in living-donor renal transplantation: a systematic review. Transplant Rev 37:100792. 10.1016/j.trre.2023.100792 10.1016/j.trre.2023.100792 37709652

[23] Spitzer TR, Delmonico F, Tolkoff-Rubin N et al (1999) Combined histocompatibility leukocyte antigenmatched donor bone marrow and renal transplantation for multiple myeloma with end stage renal disease: the induction of allograft tolerance through mixed lymphohematopoietic chimerism. Transplantation 68:480 10480403 10.1097/00007890-199908270-00006

[24] Spitzer TR, Tolkoff-Rubin N, Cosimi AB et al (2019) Twenty-year follow-up of histocompatibility leukocyte antigen-matched kidney and bone marrow cotransplantation for multiple myeloma with end-stage renal disease: lessons learned. Transplantation 103:2366. 10.1097/TP.0000000000002669 30801529 10.1097/TP.0000000000002669 PMC6690803

[25] Mapara MY, Pelot M, Zhao G et al (2001) Induction of stable long-term mixed hematopoietic chimerism following nonmyeloablative conditioning with T cell-depleting antibodies, cyclophosphamide, and thymic irradiation leads to donor-specific in vitro and in vivo tolerance. Biol Blood Marrow Transplant 7:646–655. 10.1053/bbmt.2001.v7.pm11787527 11787527 10.1053/bbmt.2001.v7.pm11787527

[26] Pelot MR, Pearson DA, Swenson K et al (1999) Lymphohematopoietic graft-vs.-host reactions can be induced without graft-vs.-host disease in murine mixed chimeras established with a cyclophosphamide-based nonmyeloablative conditioning regimen. Biol Blood Marrow Transplant 5:133–143. 10.1053/bbmt.1999.v5.pm10392959 10392959 10.1053/bbmt.1999.v5.pm10392959

[27] Kawai T, Cosimi AB, Spitzer TR et al (2008) HLA-mismatched renal transplantation without maintenance immunosuppression. N Engl J Med 358:353–361. 10.1056/NEJMoa071074 18216355 10.1056/NEJMoa071074 PMC2819046

[28] Chen Y-B, Elias N, Heher E et al (2019) Haploidentical hematopoietic cell and kidney transplantation for hematological malignancies and end-stage renal failure. Blood 134:211–215. 10.1182/blood.2019000775 31151984 10.1182/blood.2019000775 PMC6624971

[29] Kawai T, Sachs DH, Sprangers B et al (2014) Long-term results in recipients of combined HLA-mismatched kidney and bone marrow transplantation without maintenance immunosuppression. Am J Transplant 14:1599–1611. 10.1111/ajt.12731 24903438 10.1111/ajt.12731 PMC4228952

[30] Podestà MA, Binder C, Sellberg F et al (2020) Siplizumab selectively depletes effector memory T cells and promotes a relative expansion of alloreactive regulatory T cells in vitro. Am J Transplant 20:88–100. 10.1111/ajt.15533 31319439 10.1111/ajt.15533 PMC6940533

[31] Binder C, Sellberg F, Cvetkovski F, et al (2020) Siplizumab, an anti-CD2 monoclonal antibody, induces a unique set of immune modulatory effects compared to alemtuzumab and rabbit anti-thymocyte globulin in vitro. Front Immunol 11. 10.3389/fimmu.2020.592553 10.3389/fimmu.2020.592553 PMC7686512 33262770

[32] Lowsky R, Takahashi T, Liu YP et al (2005) Protective conditioning for acute graft-versus-host disease. N Engl J Med 353:1321–1331. 10.1056/NEJMoa050642 16192477 10.1056/NEJMoa050642

[33] Millan MT, Shizuru JA, Hoffmann P et al (2002) Mixed chimerism and immunosuppressive drug withdrawal after hla-mismatched kidney and hematopoietic progenitor transplantation1. Transplantation 73:1386 12023614 10.1097/00007890-200205150-00005

[34] Scandling JD, Busque S, Shizuru JA et al (2015) Chimerism, graft survival, and withdrawal of immunosuppressive drugs in HLA matched and mismatched patients after living donor kidney and hematopoietic cell transplantation. Am J Transplant 15:695–704. 10.1111/ajt.13091 25693475 10.1111/ajt.13091

[35] Busque S, Scandling JD, Lowsky R et al (2020) Mixed chimerism and acceptance of kidney transplants after immunosuppressive drug withdrawal. Sci Trans Med 12:eaax8863. 10.1126/scitranslmed.aax8863 10.1126/scitranslmed.aax8863 PMC8051148 31996467

[36] Leventhal J, Abecassis M, Miller J et al (2012) Chimerism and tolerance without GVHD or engraftment syndrome in HLA-mismatched combined kidney and hematopoietic stem cell transplantation. Sci Trans Med 4:124ra28-124ra28. 10.1126/scitranslmed.3003509 10.1126/scitranslmed.3003509 PMC3610325 22399264

[37] Gandy KL, Domen J, Aguila H, Weissman IL (1999) CD8+TCR+ and CD8+TCR− cells in whole bone marrow facilitate the engraftment of hematopoietic stem cells across allogeneic barriers. Immunity 11:579–590. 10.1016/S1074-7613(00)80133-8 10591183 10.1016/s1074-7613(00)80133-8

[38] Leventhal JR, Krieger NR, Tambur AR et al (2021) Establishment of durable chimerism with minimal GvHD in highly mismatched recipients receiving an investigational facilitated allo-HSCT. Blood 138:911. 10.1182/blood-2021-153231

[39] Fuchimoto Y, Huang CA, Yamada K et al (2000) Mixed chimerism and tolerance without whole body irradiation in a large animal model. J Clin Invest 105:1779–1789. 10.1172/JCI8721 10862793 10.1172/JCI8721 PMC378506

[40] Kawai T, Cosimi AB, Colvin RB et al (1995) Mixed allogeneic chimerism and renal allograft tolerance in cynomolgus monkeys. Transplantation 59:256 7839449

[41] Wu L, Shortman K (2005) Heterogeneity of thymic dendritic cells. Semin Immunol 17:304–312. 10.1016/j.smim.2005.05.001 15946853 10.1016/j.smim.2005.05.001

[42] Li J, Park J, Foss D, Goldschneider I (2009) Thymus-homing peripheral dendritic cells constitute two of the three major subsets of dendritic cells in the steady-state thymus. J Exp Med 206:607–622. 10.1084/jem.20082232 19273629 10.1084/jem.20082232 PMC2699131

[43] Bonasio R, Scimone ML, Schaerli P et al (2006) Clonal deletion of thymocytes by circulating dendritic cells homing to the thymus. Nat Immunol 7:1092–1100. 10.1038/ni1385 16951687 10.1038/ni1385

[44] Khan A, Tomita Y, Sykes M (1996) Thymic dependence of loss of tolerance in mixed allogeneic bone marrow chimeras after depletion of donor antigen: peripheral mechanisms do not contribute to maintenance of tolerance: 1. Transplantation 62:380 8779687 10.1097/00007890-199608150-00014

[45] Tian C, Yuan X, Bagley J et al (2008) Induction of transplantation tolerance by combining non-myeloablative conditioning with delivery of alloantigen by T cells. Clin Immunol 127:130–137. 10.1016/j.clim.2008.01.005 18280792 10.1016/j.clim.2008.01.005 PMC2430039

[46] Hakim FT, Memon SA, Cepeda R et al (2005) Age-dependent incidence, time course, and consequences of thymic renewal in adults. J Clin Invest 115:930–939. 10.1172/JCI22492 15776111 10.1172/JCI22492 PMC1064981

[47] Douek DC, McFarland RD, Keiser PH et al (1998) Changes in thymic function with age and during the treatment of HIV infection. Nature 396:690–695. 10.1038/25374 9872319 10.1038/25374

[48] Douek DC, Vescio RA, Betts MR et al (2000) Assessment of thymic output in adults after haematopoietic stem-cell transplantation and prediction of T-cell reconstitution. Lancet 355:1875–1881. 10.1016/S0140-6736(00)02293-5 10866444 10.1016/S0140-6736(00)02293-5

[49] Sho M, Kishimoto K, Harada H et al (2005) Requirements for induction and maintenance of peripheral tolerance in stringent allograft models. Proc Natl Acad Sci 102:13230–13235. 10.1073/pnas.0505070102 16150717 10.1073/pnas.0505070102 PMC1201597

[50] Zuber J, Shonts B, Lau S-P et al (2016) Bidirectional intragraft alloreactivity drives the repopulation of human intestinal allografts and correlates with clinical outcome. Sci Immunol 1:eaah3732. 10.1126/sciimmunol.aah3732 28239678 10.1126/sciimmunol.aah3732 PMC5323244

[51] Morris H, DeWolf S, Robins H et al (2015) Tracking donor-reactive T cells: evidence for clonal deletion in tolerant kidney transplant patients. Sci Transl Med 7:272ra10. 10.1126/scitranslmed.3010760 25632034 10.1126/scitranslmed.3010760 PMC4360892

[52] Tomita Y, Sachs DH, Khan A, Sykes M (1996) Additional monoclonal antibody (mAB) injections can replace thymic irradiation to allow induction of mixed chimerism and tolerance in mice receiving bone marrow transplantation after conditioning with anti-T cell mABs and 3-GY whole body irradiation1. Transplantation 61:469 8610363 10.1097/00007890-199602150-00027

[53] Cippà PE, Gabriel SS, Chen J et al (2013) Targeting apoptosis to induce stable mixed hematopoietic chimerism and long-term allograft survival without myelosuppressive conditioning in mice. Blood 122:1669–1677. 10.1182/blood-2012-09-453944 23869083 10.1182/blood-2012-09-453944

[54] Sasaki H, Hirose T, Oura T et al (2023) Selective Bcl-2 inhibition promotes hematopoietic chimerism and allograft tolerance without myelosuppression in nonhuman primates. Sci Transl Med 15:eadd5318. 10.1126/scitranslmed.add5318 37018417 10.1126/scitranslmed.add5318 PMC11022838

[55] Nadazdin O, Boskovic S, Murakami T et al (2011) Host alloreactive memory T cells influence tolerance to kidney allografts in nonhuman primates. Sci Transl Med 3:86ra51. 10.1126/scitranslmed.3002093 21653831 10.1126/scitranslmed.3002093 PMC3261229

[56] Amir AL, D’Orsogna LJA, Roelen DL et al (2010) Allo-HLA reactivity of virus-specific memory T cells is common. Blood 115:3146–3157. 10.1182/blood-2009-07-234906 20160165 10.1182/blood-2009-07-234906

[57] Lee S, Yamada Y, Tonsho M et al (2013) Alefacept promotes immunosuppression-free renal allograft survival in nonhuman primates via depletion of recipient memory T cells. Am J Transplant 13:3223–3229. 10.1111/ajt.12500 24165326 10.1111/ajt.12500 PMC4091756

[58] Yamada Y, Boskovic S, Aoyama A et al (2012) Overcoming memory T cell responses for induction of delayed tolerance in nonhuman primates. Am J Transplant 12:330–340. 10.1111/j.1600-6143.2011.03795.x 22053723 10.1111/j.1600-6143.2011.03795.x PMC3268945

[59] Braza F, Dugast E, Panov I et al (2015) Central role of CD45RA− foxp3hi memory regulatory T cells in clinical kidney transplantation tolerance. J Am Soc Nephrol 26:1795–1805. 10.1681/ASN.2014050480 25556168 10.1681/ASN.2014050480 PMC4520169

[60] Sprangers B, DeWolf S, Savage TM et al (2017) Origin of enriched regulatory T cells in patients receiving combined kidney-bone marrow transplantation to induce transplantation tolerance. Am J Transplant 17:2020–2032. 10.1111/ajt.14251 28251801 10.1111/ajt.14251 PMC5519438

[61] Savage TM, Shonts BA, Obradovic A et al (2018) Early expansion of donor-specific Tregs in tolerant kidney transplant recipients. JCI Insight 3. 10.1172/jci.insight.124086 10.1172/jci.insight.124086 PMC6302945 30429370

[62] Hotta K, Aoyama A, Oura T et al (2019) Induced regulatory T cells in allograft tolerance via transient mixed chimerism. JCI Insight 1:e86419. 10.1172/jci.insight.86419 10.1172/jci.insight.86419 PMC4951099 27446989

[63] Kendal AR, Chen Y, Regateiro FS et al (2011) Sustained suppression by Foxp3+ regulatory T cells is vital for infectious transplantation tolerance. J Exp Med 208:2043–2053. 10.1084/jem.20110767 21875958 10.1084/jem.20110767 PMC3182049

[64] Gravano DM, Vignali DAA (2012) The battle against immunopathology: infectious tolerance mediated by regulatory T cells. Cell Mol Life Sci 69:1997–2008. 10.1007/s00018-011-0907-z 22205213 10.1007/s00018-011-0907-z PMC3353028

[65] Matsuoka K, Koreth J, Kim HT et al (2013) Low-dose interleukin-2 therapy restores regulatory T cell homeostasis in patients with chronic graft-versus-host disease. Sci Trans Med 5:179ra43-179ra43. 10.1126/scitranslmed.3005265 10.1126/scitranslmed.3005265 PMC3686517 23552371

[66] Yamada Y, Nadazdin O, Boskovic S et al (2015) Repeated injections of IL-2 break renal allograft tolerance induced via mixed hematopoietic chimerism in monkeys. Am J Transplant 15:3055–3066. 10.1111/ajt.13382 26190648 10.1111/ajt.13382 PMC4654979

[67] Schoenbrunn A, Frentsch M, Kohler S et al (2012) A converse 4–1BB and CD40 ligand expression pattern delineates activated regulatory T cells (Treg) and conventional T cells enabling direct isolation of alloantigen-reactive natural foxp3+ treg. J Immunol 189:5985–5994. 10.4049/jimmunol.1201090 23162126 10.4049/jimmunol.1201090

[68] Poirier N, Azimzadeh AM, Zhang T et al (2010) Inducing CTLA-4–dependent immune regulation by selective CD28 blockade promotes regulatory T cells in organ transplantation. Sci Trans Med 2:17ra10-17ra10. 10.1126/scitranslmed.3000116 10.1126/scitranslmed.3000116 PMC2860737 20371478

[69] Wekerle T, Sayegh MH, Hill J et al (1998) Extrathymic T cell deletion and allogeneic stem cell engraftment induced with costimulatory blockade is followed by central T cell tolerance. J Exp Med 187:2037–2044 9625763 10.1084/jem.187.12.2037 PMC2212372

[70] Pilat N, Baranyi U, Klaus C et al (2010) Treg-therapy allows mixed chimerism and transplantation tolerance without cytoreductive conditioning. Am J Transplant 10:751–762. 10.1111/j.1600-6143.2010.03018.x 20148810 10.1111/j.1600-6143.2010.03018.x PMC2856406

[71] Sagoo P, Ali N, Garg G et al (2011) Human regulatory T cells with alloantigen specificity are more potent inhibitors of alloimmune skin graft damage than polyclonal regulatory T cells. Sci Trans Med 3:83ra42-83ra42. 10.1126/scitranslmed.3002076 10.1126/scitranslmed.3002076 PMC3776382 21593402

[72] MacDonald KG, Hoeppli RE, Huang Q et al (2016) Alloantigen-specific regulatory T cells generated with a chimeric antigen receptor. J Clin Invest 126:1413–1424. 10.1172/JCI82771 26999600 10.1172/JCI82771 PMC4811124

[73] Oberbauer R, Edinger M, Berlakovich G et al (2021) A prospective controlled trial to evaluate safety and efficacy of in vitro expanded recipient regulatory T cell therapy and tocilizumab together with donor bone marrow infusion in HLA-mismatched living donor kidney transplant recipients (Trex001). Front Med 7. 10.3389/fmed.2020.634260 10.3389/fmed.2020.634260 PMC7873436 33585521

[74] McCallion O, Bilici M, Hester J, Issa F (2022) Regulatory T-cell therapy approaches. Clin Exp Immunol 211:96–107. 10.1093/cei/uxac078 10.1093/cei/uxac078 PMC10019137 35960852

[75] Pilat N, Steiner R, Sprent J (2023) Treg therapy for the induction of immune tolerance in transplantation-not lost in translation? Int J Mol Sci 24:1752. 10.3390/ijms24021752 36675265 10.3390/ijms24021752 PMC9861925

[76] Zuber J, Rosen S, Shonts B et al (2015) Macrochimerism in intestinal transplantation: association with lower rejection rates and multivisceral transplants, without GVHD. Am J Transplant 15:2691–2703. 10.1111/ajt.13325 25988811 10.1111/ajt.13325 PMC4575629

[77] Mapara MY, Kim Y-M, Wang S-P et al (2002) Donor lymphocyte infusions mediate superior graft-versus-leukemia effects in mixed compared to fully allogeneic chimeras: a critical role for host antigen-presenting cells. Blood 100:1903–1909. 10.1182/blood-2002-01-0023 12176915 10.1182/blood-2002-01-0023

[78] Chakraverty R, Côté D, Buchli J et al (2006) An inflammatory checkpoint regulates recruitment of graft-versus-host reactive T cells to peripheral tissues. J Exp Med 203:2021–2031. 10.1084/jem.20060376 16880259 10.1084/jem.20060376 PMC2118376

[79] Sykes M (2024) Tolerance in intestinal transplantation. Hum Immunol 85:110793. 10.1016/j.humimm.2024.110793 38580539 10.1016/j.humimm.2024.110793 PMC11144570

[80] Fu J, Zuber J, Shonts B et al (2021) Lymphohematopoietic graft-versus-host responses promote mixed chimerism in patients receiving intestinal transplantation. J Clin Invest 131(e141698):141698. 10.1172/JCI141698 33630757 10.1172/JCI141698 PMC8062082

[81] Cómitre-Mariano B, Martínez-García M, García-Gálvez B et al (2021) Feto-maternal microchimerism: memories from pregnancy. iScience 25:103664. 10.1016/j.isci.2021.103664 35072002 10.1016/j.isci.2021.103664 PMC8762399

[82] Stelzer IA, Thiele K, Solano ME (2015) Maternal microchimerism: lessons learned from murine models. J Reprod Immunol 108:12–25. 10.1016/j.jri.2014.12.007 25638482 10.1016/j.jri.2014.12.007

[83] Stelzer IA, Urbschat C, Schepanski S et al (2021) Vertically transferred maternal immune cells promote neonatal immunity against early life infections. Nat Commun 12:4706. 10.1038/s41467-021-24719-z 34349112 10.1038/s41467-021-24719-z PMC8338998

[84] Hall J, Lingenfelter P, Adams S et al (1995) Detection of maternal cells in human umbilical cord blood using fluorescence in situ hybridization. Blood 86:2829–2832. 10.1182/blood.V86.7.2829.2829 7545474

[85] Kinder JM, Stelzer IA, Arck PC, Way SS (2017) Immunological implications of pregnancy-induced microchimerism. Nat Rev Immunol 17:483–494. 10.1038/nri.2017.38 28480895 10.1038/nri.2017.38 PMC5532073

[86] Fujimoto K, Nakajima A, Hori S et al (2022) Whole-embryonic identification of maternal microchimeric cell types in mouse using single-cell RNA sequencing. Sci Rep 12:18313. 10.1038/s41598-022-20781-9 36333354 10.1038/s41598-022-20781-9 PMC9636240

[87] Vanzyl B, Planas R, Ye Y et al (2010) Why are levels of maternal microchimerism higher in type 1 diabetes pancreas? Chimerism 1:45–50. 10.4161/chim.1.2.13891 21327046 10.4161/chim.1.2.13891 PMC3023622

[88] Muraji T, Hosaka N, Irie N et al (2008) Maternal microchimerism in underlying pathogenesis of biliary atresia: quantification and phenotypes of maternal cells in the liver. Pediatrics 121:517–521. 10.1542/peds.2007-0568 18310200 10.1542/peds.2007-0568

[89] Graf I, Urbschat C, Arck PC (2024) The “communicatome” of pregnancy: spotlight on cellular and extravesicular chimerism. EMBO Mol Med 16:700–714. 10.1038/s44321-024-00045-x 38467841 10.1038/s44321-024-00045-x PMC11018796

[90] Wegorzewska M, Le T, Tang Q, MacKenzie TC (2014) Increased maternal T cell microchimerism in the allogeneic fetus during LPS-induced preterm labor in mice. Chimerism 5:68–74. 10.1080/19381956.2014.1002703 25779065 10.1080/19381956.2014.1002703 PMC5063068

[91] Balle C, Armistead B, Kiravu A et al (2022) Factors influencing maternal microchimerism throughout infancy and its impact on infant T cell immunity. J Clin Invest 132:e148826. 10.1172/JCI148826 35550376 10.1172/JCI148826 PMC9246390

[92] Mold JE, Michaëlsson J, Burt TD et al (2008) Maternal alloantigens promote the development of tolerogenic fetal regulatory T cells in utero. Science 322:1562–1565. 10.1126/science.1164511 19056990 10.1126/science.1164511 PMC2648820

[93] Przybyszewski EM, Verna EC, Lobritto SJ et al (2018) Durable clinical and immunologic advantage of living donor liver transplantation in children. Transplantation 102:953. 10.1097/TP.0000000000002110 29369249 10.1097/TP.0000000000002110

[94] Sanada Y, Kawano Y, Miki A et al (2014) Maternal grafts protect daughter recipients from acute cellular rejection after pediatric living donor liver transplantation for biliary atresia. Transpl Int 27:383–390. 10.1111/tri.12273 24472036 10.1111/tri.12273

[95] Nijagal A, Fleck S, Hills NK et al (2012) Decreased risk of graft failure with maternal liver transplantation in patients with biliary atresia. Am J Transplant 12:409–419. 10.1111/j.1600-6143.2011.03895.x 22221561 10.1111/j.1600-6143.2011.03895.x

[96] Gurevich M, Guy-Viterbo V, Janssen M et al (2015) Living donor liver transplantation in children: surgical and immunological results in 250 Recipients at Université Catholique de Louvain. Ann Surg 262:1141. 10.1097/SLA.0000000000001094 25563870 10.1097/SLA.0000000000001094

[97] Barbetta A, Meeberg G, Rocque B et al (2022) Immunologic benefits of maternal living donor allografts in pediatric liver transplantation: fewer rejection episodes and no evidence of de novo allosensitization. Pediatr Transplant 26:e14197. 10.1111/petr.14197 34806273 10.1111/petr.14197 PMC9053650

[98] Masuya R, Muraji T, Harumatsu T et al (2022) Biliary atresia: graft-versus-host disease with maternal microchimerism as an etiopathogenesis. Transfus Apher Sci 61. 10.1016/j.transci.2022.103410 10.1016/j.transci.2022.103410 35288054

[99] Kobayashi H, Tamatani T, Tamura T et al (2007) Maternal microchimerism in biliary atresia. J Pediatr Surg 42:987–991. 10.1016/j.jpedsurg.2007.01.051 17560207 10.1016/j.jpedsurg.2007.01.051

[100] Engels G, Döhler B, Tönshoff B et al (2022) Maternal versus paternal living kidney transplant donation is associated with lower rejection in young pediatric recipients: a collaborative transplant study report. Pediatr Transplant 26:e14154. 10.1111/petr.14154 34612565 10.1111/petr.14154

[101] Burlingham WJ, Grailer AP, Heisey DM et al (1998) The effect of tolerance to noninherited maternal HLA antigens on the survival of renal transplants from sibling donors. N Engl J Med 339:1657–1664. 10.1056/NEJM199812033392302 9834302 10.1056/NEJM199812033392302

[102] Joo SY, Song EY, Shin Y et al (2013) Beneficial effects of pretransplantation microchimerism on rejection-free survival in HLA-haploidentical family donor renal transplantation. Transplantation 95:1375–1382. 10.1097/TP.0b013e31828b10a1 23519024 10.1097/TP.0b013e31828b10a1

[103] Neu AM, Stablein DM, Zachary A et al (1998) Effect of parental donor sex on rejection in pediatric renal transplantation: a report of the North American Pediatric Renal Transplant Cooperative Study. Pediatr Transplant 2:309–312 10084735

[104] Miles CD, Schaubel DE, Liu D et al (2008) The role of donor-recipient relationship in long-term outcomes of living donor renal transplantation. Transplantation 85:1483. 10.1097/TP.0b013e3181705a0f 18497690 10.1097/TP.0b013e3181705a0f

[105] Haynes WJ, Jankowska-Gan E, Haynes L, Burlingham WJ (2014) Microchimerism and regulation in living related kidney transplant families. Chimerism 5:80–85. 10.1080/19381956.2015.1111974 26679771 10.1080/19381956.2015.1111974 PMC5063072

[106] Lim WH, McDonald SP, Coates PT et al (2016) Maternal compared with paternal donor kidneys are associated with poorer graft outcomes after kidney transplantation. Kidney Int 89:659–665. 10.1016/j.kint.2015.11.016 26880459 10.1016/j.kint.2015.11.016

[107] Montano-Loza AJ, Rodríguez-Perálvarez ML, Pageaux G-P et al (2023) Liver transplantation immunology: Immunosuppression, rejection, and immunomodulation. J Hepatol 78:1199–1215. 10.1016/j.jhep.2023.01.030 37208106 10.1016/j.jhep.2023.01.030

[108] Kim J-Y, Kim HB, Kim J-M, et al (2024) Immunoprotective effect of liver allograft on patients with combined liver and kidney transplantation. Ann Transplant 29:e942763–1-e942763–11. 10.12659/AOT.942763 10.12659/AOT.942763 PMC10858615 38319291

[109] Ichinohe T, Teshima T, Matsuoka K et al (2005) Fetal–maternal microchimerism: impact on hematopoietic stem cell transplantation. Curr Opin Immunol 17:546–552. 10.1016/j.coi.2005.07.009 16084712 10.1016/j.coi.2005.07.009

[110] van Rood JJ, Scaradavou A, Stevens CE (2012) Indirect evidence that maternal microchimerism in cord blood mediates a graft-versus-leukemia effect in cord blood transplantation. Proc Natl Acad Sci 109:2509–2514. 10.1073/pnas.1119541109 22232664 10.1073/pnas.1119541109 PMC3289309

[111] Osada H, Doi S, Fukushima T et al (2001) Detection of fetal HPCs in maternal circulation after delivery. Transfusion 41:499–503. 10.1046/j.1537-2995.2001.41040499.x 11316901 10.1046/j.1537-2995.2001.41040499.x

[112] Shao T-Y, Kinder JM, Harper G et al (2023) Reproductive outcomes after pregnancy-induced displacement of preexisting microchimeric cells. Science 381:1324–1330. 10.1126/science.adf9325 37733857 10.1126/science.adf9325 PMC10877202

[113] Gillis-Buck EM, Gardner JM (2024) Maternal-fetal microchimerism as a durable but finite and replaceable alloantigen reservoir. Am J Transplant 24:512–513. 10.1016/j.ajt.2024.03.002 38556430 10.1016/j.ajt.2024.03.002

[114] Suah AN, Tran D-KV, Khiew SHW et al (2021) Pregnancy-induced humoral sensitization overrides T cell tolerance to fetus-matched allografts in mice. J Clin Invest 131. 10.1172/JCI140715 10.1172/JCI140715 PMC7773355 33393512

[115] Manook M, Olaso D, Anwar IJ et al (2023) Desensitization and belatacept-based maintenance therapy in pregnancy-sensitized monkeys receiving a kidney transplant. Sci Adv 9:eadg1448. 10.1126/sciadv.adg1448 37205758 10.1126/sciadv.adg1448 PMC10198638

